# The immunogenicity and safety of a reduced PRP-content DTPw-HBV/Hib vaccine when administered according to the accelerated EPI schedule

**DOI:** 10.1186/1471-2334-10-298

**Published:** 2010-10-15

**Authors:** Sukanta Chatterjee, Sylvan J Rego, Fulton D'Souza, BD Bhatia, Alix Collard, Sanjoy K Datta, Jeanne-Marie Jacquet

**Affiliations:** 1 Department of Pediatrics, Medical College Kolkata, Kolkata, India; 2 St John's Medical College Hospital, Bangalore, India; 3 Department of Pediatrics, Institute of Medical Sciences, Banaras Hindu University, Varanasi, India; 4 GlaxoSmithKline Biologicals, Bangalore, India and Wavre, Belgium

## Abstract

**Background:**

Combination vaccines improve coverage, compliance and effectively introduce new antigens to mass vaccination programmes. This was a phase III, observer-blind, randomized study of GSK Biologicals diphtheria-tetanus-whole cell pertussis vaccine combined with hepatitis B and *Haemophilus influenzae *type b vaccines, containing a reduced amount of polyribosyl-ribitol-phosphate (PRP) and a DTPw component manufactured at a different site (DTPw-HBV/Hib_2.5 _[Kft]). The primary aim of this study was to demonstrate that DTPw-HBV/Hib_2.5 _[Kft] was not inferior to the licensed DTPw-HBV/Hib (*Tritanrix*(tm)-HepB/*Hiberix*(tm)) vaccine or the DTPw-HBV/Hib_2.5 _vaccine, also containing a reduced amount of PRP, with respect to the immune response to the PRP antigen, when administered to healthy infants, according to the Expanded Programme for Immunization (EPI) schedule at 6, 10 and 14 weeks of age.

**Methods:**

299 healthy infants were randomised to receive either DTPw-HBV/Hib_2.5 _[Kft] DTPw-HBV/Hib_2.5 _or DTPw-HBV/Hib according to the 6-10-14 week EPI schedule. Blood samples were analysed prior to the first dose of study vaccine and one month after the third vaccine dose for the analysis of immune responses. Solicited local and general symptoms such as pain, redness and swelling at the injection site and drowsiness and fever, unsolicited symptoms (defined as any additional adverse event) and serious adverse events (SAEs) were recorded up to 20 weeks of age.

**Results:**

One month after the third vaccine dose, 100% of subjects receiving DTPw-HBV/Hib_2.5 _[Kft] or DTPw-HBV/Hib and 98.8% of subjects receiving DTPw-HBV/Hib_2.5 _vaccine had seroprotective levels of anti-PRP antibodies (defined as anti-PRP antibody concentration ≥0.15 μg/ml). Seroprotective antibody concentrations were attained in over 98.9% of subjects for diphtheria, tetanus and hepatitis B. The vaccine response rate to pertussis antigen was at least 97.8% in each group. Overall, the DTPw-HBV/Hib_2.5 _[Kft] vaccine was well tolerated in healthy infants; no SAEs were reported in any group.

**Conclusions:**

The DTPw-HBV/Hib_2.5 _[Kft] vaccine was immunogenic and well-tolerated when administered according to the EPI schedule to Indian infants.

**Trial registration:**

http://www.clinicaltrials.gov NCT00473668

## Background

Combination vaccines improve individual compliance and vaccination coverage and offer a convenient vehicle for introducing community protection against new diseases by adding antigens to an existing vaccine with high coverage [1,2]. The diphtheria-tetanus-whole cell pertussis (DTPw) is one such vaccine, with a global coverage of 81% in 2007 [3].

In 1996, GlaxoSmithKline (GSK) Biologicals licensed the first combined DTPw and hepatitis B vaccine (DTPw-HBV, *Tritanrix*(tm)Hep B), which was shown to improve the uptake of hepatitis B vaccine in Thailand [4]. *Haemophilus influenzae *type b (Hib) protection was added via the monovalent vaccine, *Hiberix*(tm) to form DTPw-HBV/Hib. This combination vaccine facilitated the introduction of hepatitis B and Hib vaccinations to large parts of the developing world [5].

Increasing global demands for DTPw-based combination vaccines has necessitated creative strategies to ensure the adequate supply of the vaccine antigens, through reducing Hib antigen content and expanding antigen production at new manufacturing sites. Primary vaccination with reduced Hib content vaccines has been shown to confer vaccine response rates, after primary vaccination, at least as high as those observed with commercially available DTPw-HBV/Hib containing 10 μg PRP [6,7]. DTPw-HBV/Hib_2.5_, which contains a reduced amount of the purified polyribosyl-ribitol-phosphate capsular polysaccharide (PRP) of Hib covalently bound to tetanus toxoid has been subsequently developed. Furthermore, a formulation of this vaccine [(DTPw-HBV/Hib_2.5 _[Kft]; *Zilbrix*(tm)-Hib)] manufactured at a new production site in Hungary and represented by 'Kft' has been introduced.

The primary aim of this study was to demonstrate that (DTPw-HBV/Hib_2.5 _[Kft]) was not inferior to the licensed DTPw-HBV/Hib (*Tritanrix*(tm)-HepB/*Hiberix*(tm)) vaccine or the DTPw-HBV/Hib_2.5 _vaccine with respect to the immune response to the PRP antigen, when administered to healthy infants according to the Expanded Programme for Immunization (EPI) schedule at 6, 10 and 14 weeks of age. The DTPw-HBV/Hib_2.5 _[Kft] differs from the other two vaccines in the study as it has components manufactured at a site in Hungary.

## Methods

### Study design and subjects

This phase III, observer-blind, randomized, primary vaccination study took place at three centres in India between June 2007 and January 2008. The trial followed the principles of the Declaration of Helsinki, and was compliant with the international standards of Good Clinical Practice and the local Indian Council of Medical Research guidelines governing clinical trials [8]. The study protocol was approved by the office of the Drugs Controller General of India (DCGI). The protocol was also subject to an institutional ethics committee review at each centre. Additionally, the study processes were subject to a sponsor audit without any critical findings.

Written, informed consent was obtained from parents/guardians before enrolment. Healthy infants aged 6-8 weeks who had received one dose of the Hep B vaccine within one week of birth were randomised to receive the DTPw-HBV/Hib_2.5 _[Kft], DTPw-HBV/Hib_2.5_ or DTPw-HBV/Hib vaccines at 6, 10 and 14 weeks of age by intramuscular injection in the thigh.

### Vaccines

The diphtheria and tetanus antigens of the DTPw-HBV/Hib_2.5 _[Kft] vaccine were produced at GSK Biologicals, Korlatolt Felelossegu Tarsasag in Hungary, all other components of this, and the other vaccines, were developed and manufactured by GSK Biologicals, Rixensart, Belgium. The pertussis components were produced by the Commonwealth Serum Laboratory in Australia.

All vaccines contained: at least 30 international units (IU) of diphtheria toxoid, 60 IU of tetanus toxoid and 4 IU of *Bordetella pertussis *(BPT), killed; and 10 μg of hepatitis B surface antigen (HBsAg). The study vaccine, DTPw-HBV/Hib_2.5 _[Kft] and the comparator DTPw-HBV/Hib_2.5 _both contained 2.5 μg of the *H. influenzae *type b capsular polysaccharide conjugated to 5-10 μg of the tetanus toxoid, compared to 10 μg of the *H. influenzae *type b capsular polysaccharide conjugated to 20 to 40 μg tetanus toxoid in the DTPw-HBV/Hib vaccine.

### Assessment of immunogenicity

Blood samples were collected before the first dose of study vaccine and one month after the third vaccine dose and were tested for antibodies against all vaccine antigens, using enzyme-linked immunosorbent assays (ELISAs).

Anti-PRP antibodies were measured by ELISA with a cut-off set at 0.15 μg/ml. Anti-diphtheria and anti-tetanus antibody concentrations were measured by ELISA with an assay cut-off set at 0.10 IU/ml. Subjects seronegative for anti-diphtheria antibodies by ELISA were re-tested with an in vitro neutralization assay on Vero cells (cut-off of 0.016 IU/ml). Anti-HBs antibodies were determined using either a commercial radioimmunoassay (AUSAB, Abbott), an ELISA developed in house or combination of the two, with an assay cut-off set at 10 mIU/ml. Seroprotection was defined as antibody concentrations greater or equal to the assay cut-off. Anti-whole-cell-*Bordetella Pertussis *(BPT) antibody concentrations were measured by ELISA (AniLabsystems) with an assay cut-off set at 15 ELU/ml.

### Assessment of Reactogenicity

Reactogenicity was assessed using diary cards during a 4-day follow-up period after each vaccination. Reports of local symptoms of pain, redness and swelling at the site of injection, and of general symptoms of drowsiness, fever (defined as an axillary temperature ≥37.5°C), irritability and loss of appetite were actively solicited. Symptom intensities for pain, irritability, drowsiness and loss of appetite were graded by the investigators on a three point scale. Grade 3 was defined as: cries when limb is moved/spontaneously painful (pain); diameter > 20 mm (swelling and redness); interfering with normal activities (other symptoms).

Unsolicited symptoms were recorded during the 30-day follow-up period after each vaccine dose and serious adverse events (SAE) were recorded throughout the duration of the trial.

### Statistical Analysis

The primary objective of the study was to sequentially assess and check the non-inferiority of the DTPw-HBV/Hib_2.5 _[Kft] vaccine versus first DTPw-HBV/Hib and then DTPw-HBV/Hib_2.5_, in terms of the anti-PRP antibody response, after a three-dose primary vaccination course administered to healthy infants at 6, 10 and 14 weeks of age.

The analysis of immunogenicity was based on the according to protocol (ATP) cohort for analysis of immunogenicity, which included all evaluable subjects for whom at least one measurement for the immunogenicity endpoint measures were available and who followed the study procedures. Evaluable subjects were those that met all the eligibility criteria, complied with the procedures defined in the protocol and were not eliminated from the study.

It was calculated that a sample size of 90 evaluable subjects per group would provide an overall power of 92% to meet the non-inferiority criteria for both the primary endpoints assuming the groups elicited identical immune responses. Allowing for an attrition rate of 10%, a target sample size of 300 subjects (100 subjects per vaccine group) was selected.

Antibody seroprotection rates were calculated with 95% confidence intervals (CIs) one month after the third vaccine dose. Geometric mean concentrations (GMCs) were calculated by taking the anti-log of the mean of the log concentration transformations. Antibody concentrations below the cut-off of the assay were given an arbitrary value of half the cut-off for the purpose of GMC calculation. As there is no established correlate of protection against pertussis, a vaccine response was defined as the appearance of antibodies in initially seronegative subjects, or maintenance or increase of pre-vaccination antibody concentrations in subjects that were seropositive prior to vaccination, taking into account the decline of maternal antibodies. All CIs calculated were 2-sided and computed using Proc StatXact SAS 9.1 and Proc StatXact 5 procedure on SAS. Non-inferiority was considered to be reached if the upper limit of the two-sided 95% CI, for the differences in the percentage of subjects with anti-PRP antibody concentrations of ≥0.15 μg/ml, was below the pre-defined limit of 10% one month after the 3^rd ^vaccine dose. Additional exploratory analyses of seroprotection using 95% CI on differences in seroprotection or vaccine response rates or ratios of GMCs between the study vaccine and comparator groups were undertaken.

The safety analysis was based on the Total Vaccinated Cohort. The incidence of solicited local and general adverse events (any or grade 3 intensity) was calculated using exact 95% CI. Exploratory analyses were used to compare the incidence of solicited symptoms between the groups. The standardized asymptotic 95% CI for the difference between groups was also computed (StatXact 7) for the occurrence of solicited symptoms (during the 4-day follow-up period) after each vaccine dose, and overall per dose and per subject. CIs excluding zero indicated that a difference between groups may exist.

## Results

**Figure 1:**
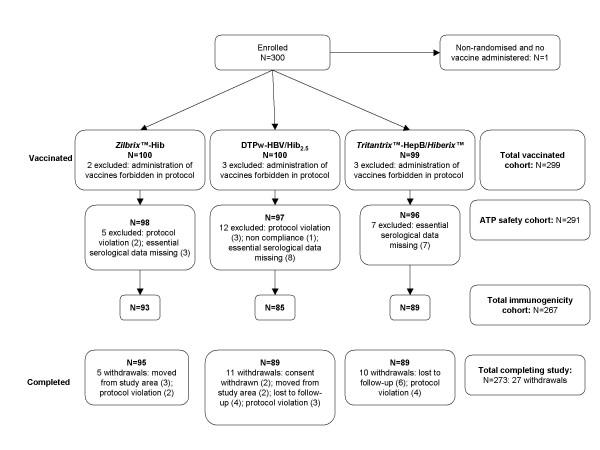
**Distribution of subjects (CONSORT diagram)**.

A total of 300 subjects were recruited, of whom 299 subjects were vaccinated and included in the total vaccinated cohort and 267 were included in the ATP immunogenicity cohort of subjects (Figure 1). There were no withdrawals due to adverse events and 273 subjects completed the study. The demographic characteristics of the subjects in the three groups were similar (Table 1).

**Table 1:** Summary of demographic characteristics (Total Vaccinated Cohort)

		**DTPw-HBV/Hib**_**2.5 **_**[Kft] (N = 100)**		DTPw-HBV/Hib (N = 99)		DTPw-HBV/Hib2.5 (N = 100)		Total (N = 299)	
Characteristics		Value or n	%	Value or n	%	Value or n	%	Value or n	%
Age (weeks)	Mean	6.3	-	6.3	-	6.4	-	6.3	-
	SD	0.59	-	0.51	-	0.64	-	0.58	-
Weight (kg)	Mean	3.9	-	3.9	-	3.8	-	3.9	-
	SD	0.69	-	0.66	-	0.70	-	0.69	-
Gender	Female	39	39.0	37	37.4	48	48.0	124	41.5
	Male	61	61.0	62	62.6	52	52.0	175	58.5
Race	Asian	100	100.0	99	100.0	100	100.0	299	100.0

N = total number of subjects; n/% = number/percentage of subjects in a given category; SD = standard deviation

### Immunogenicity

One month after the third vaccine dose, all subjects vaccinated with DTPw-HBV/Hib_2.5 _[Kft] and DTPw-HBV/Hib and 98.8% of those vaccinated with DTPw-HBV/Hib_2.5 _had seroprotective levels of anti-PRP antibodies across all three groups (Table 2). The upper limit of the 95% CI for the difference between groups in the percentage of subjects with seroprotective anti-PRP antibody concentrations (≥0.15 μg/ml) was below the pre-defined limit of 10%. Thus the study vaccine, DTPw-HBV/Hib_2.5 _[Kft], was shown to be non-inferior to the two comparator vaccines.

**Table 2:** Anti-PRP antibody response before dose 1 (pre) and one month post-dose 3 (post; ATP cohort for immunogenicity)

	Time point		≥0.15 μg/mL		≥1.0 μg/mL		GMC	
		N	%	95% CI	%	95% CI	Value	95% CI
**DTPw-HBV/Hib_2.5 _[Kft]**	Pre	93	44.1	33.8-54.8	9.7	4.5-17.6	0.179	0.139-0.230
	Post	93	100	96.1-100	94.6	87.9-98.2	26.71	19.161-37.23
**DTPw-HBV/Hib**	Pre	89	42.7	32.3-53.6	10.1	4.7-18.3	0.177	0.137-0.227
	Post	89	100	95.9-100	100	95.9-100	40.75	32.301-51.40
**DTPw-HBV/Hib_2.5_**	Pre	84	42.9	32.1-54.1	8.3	3.4-16.4	0.167	0.131-0.212
	Post	85	98.8	93.6-100	94.1	86.8-98.1	19.58	14.019-27.36

N = total number of subjects; CI = confidence interval; GMC = geometric mean concentration.

In all three groups, seroprotective antibody concentrations were attained in 100% of subjects for diphtheria, tetanus and hepatitis B one month after the vaccination course. The vaccine response rate to the pertussis component, at the same time point, was at least 97.8% in each group (Table 3).

**Table 3:** Seroprotection rates and GMCs for diphtheria, tetanus, HBV and pertussis before dose 1 (pre) and one month post-dose 3 (post; ATP cohort for immunogenicity)

		Seroprotection rates/Vaccine response rates									GMC					
		***DTPw-HBV/Hib**_**2.5 **_**[Kft]***			***DTPw-HBV/Hib***			***DTPw*-HBV/Hib**_**2.5**_			***DTPw-HBV/Hib**_**2.5 **_**[Kft]***		***DTPw-HBV/Hib***		***DTPw*-HBV/Hib**_**2.5**_	
	Time point	N	%	95% CI	N	%	95% CI	N	%	95% CI	Value	95% CI	Value	95% CI	Value	95% CI
**Diphtheria ≥0.1 IU/ml **(or ≥0.016 IU/ml by neutralization assay)	Pre	93	31.2	22.0-41.6	89	34.8	25.0-45.7	85	24.7	16.0-35.3	0.086	0.070-0.105	0.082	0.069-0.097	0.076	0.063-0.093
	Post	93	100	96.1-100	89	98.9	95.9-100	85	100	95.8-100	2.89	2.30-3.64	1.76	1.41-2.19	1.65	1.36-2.02
**Tetanus ≥0.1 IU/ml**	Pre	93	100	96.1-100	89	100	95.9-100	85	100	95.8-100	1.950	1.561-2.437	2.416	1.991-2.932	2.112	1.751-2.548
	Post	93	100	96.1-100	89	100	95.9-100	85	100	95.8-100	4.74	3.78-5.95	2.82	2.32-3.45	2.77	2.24-3.44
**HBV ≥10 mIU/ml**	Pre	85	25.9	17.0-36.5	85	24.7	16.0-35.3	84	21.4	13.2-31.7	8.3	6.7-10.3	8.0	6.5-9.8	6.6	5.8-7.5
	Post	92	100	96.1-100	89	100	95.9-100	85	100	95.8-100	781.1	629.7-968.8	598.2	477.7-749.1	695.3	542.1-891.7
**Anti-BPT antibodies ≥15 El.U/ml**	Pre	93	16.1	9.3-25.2	89	19.1	11.5-28.8	85	22.4	14.0-32.7	9.1	8.2-10.1	9.1	8.3-10.0	9.8	8.7-11.0
	Post	93	98.9	94.2-100	89	98.9	93.9-100	85	100	95.8-100	63.4	55.4-72.7	83.7	73.6-95.2	100.3	88.4-113.7
**VR for anti-BPT antibody**	Post	93	97.8	92.4-99.7	89	98.9	93.9-100	85	98.8	93.6-100						

BPT = *Bordetella pertussis*; CI = confidence interval; GMC = geometric mean concentration; HBV = hepatitis B vaccine; IU = international unit; N = number of subjects with available results; VR = vaccine response.

Additional exploratory analyses also indicated the non-inferiority of DTPw-HBV/Hib_2.5 _[Kft] to DTPw-HBV/Hib and DTPw-HBV/Hib_2.5 _in terms of seroprotection rates to diphtheria, tetanus and HepB antigens and of vaccine response to the pertussis component. Non-inferiority of DTPw-HBV/Hib_2.5 _[Kft] to DTPw-HBV/Hib_2.5 _was also demonstrated for antibody GMCs to diphtheria, tetanus, HepB and PRP antigens and for DTPw-HBV/Hib_2.5 _[Kft] compared to DTPw-HBV/Hib with respect to antibody GMCs to the diphtheria, tetanus and hepatitis B antigens.

### Safety

**Figure 2:**
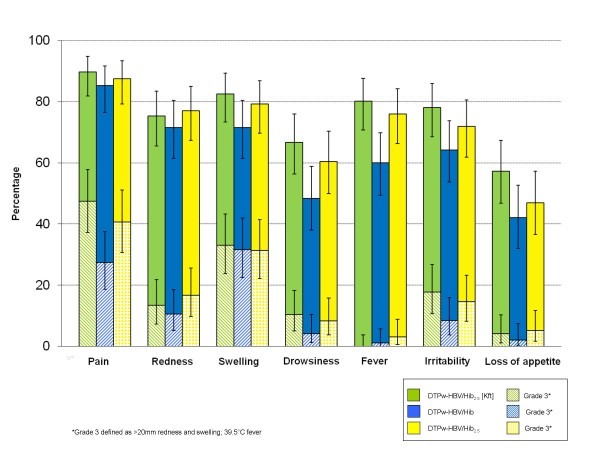
**Incidence of solicited local and general adverse events, within 4 days of vaccination after all doses (Total Vaccinated Cohort)**.

Pain was the most frequently observed local solicited symptom during the 4-day follow-up period (Figure 2). Analysis per subject showed that fever was the most frequently observed solicited general symptom for DTPw-HBV/Hib_2.5 _[Kft] (80.2%) and DTPw-HBV/Hib_2.5 _(76.0%) subjects, while irritability was the most frequently observed solicited general symptom for DTPw-HBV/Hib (64.2%). A higher overall incidence of grade 3 adverse events was observed in the DTPw-HBV/Hib_2.5 _[Kft] group.

The percentage of subjects reporting at least one unsolicited symptom within the 31 day follow up period after the administration of any vaccination dose was less than 6% in any of the groups. None of the subjects reported grade 3 unsolicited symptoms related to vaccination.

No SAEs were reported in any of the groups.

## Discussion

Vaccination of Indian infants against diphtheria, tetanus and pertussis is recommended according to the EPI schedule at 6, 10 and 14 weeks [9]. In addition, the Indian Academy of Pediatrics and WHO [10] recommend a birth dose of HBV to reduce the vertical transmission of hepatitis B to newborns, as well as HBV priming doses at the EPI time points. In 2006, WHO recommended the worldwide incorporation of *Haemophilus influenzae *type b vaccination into all routine infant immunization programs from as early as possible after 6 weeks of age [11]. In India Hib priming doses at the EPI time points are recommended.

Co-administration of multiple antigens in a single injection is widely accepted as beneficial with respect to convenience, compliance and timeliness of vaccination [1,2,12]. With respect to the DTPw-HBV-Hib vaccines, many clinical studies have shown that the five antigens can be co-administered without impacting upon the immunogenicity of the individual components [13]. The combination vaccine, DTPw-HBV-Hib, containing 10 μg PRP was licensed for primary and booster immunization of Indian infants in 2000 and has been locally shown to be immunogenic and well tolerated [14].

This trial studied the administration of the DTPw-HBV/Hib_2.5 _[Kft] vaccine according to the challenging 6-10-14 week EPI schedule, with prior administration of a birth dose of HBV, as recommended locally by the IAP and the WHO.

All subjects were seroprotected against Hib, diphtheria, tetanus and HBV one month after the third dose and 97.8% subjects showed a vaccine response to pertussis. These findings are similar to those achieved by the licensed vaccine, and comparable to levels previously reported in studies using these pentavalent vaccines in an Asian population [5,6,14-16]. Our study demonstrated DTPw-HBV/Hib_2.5 _[Kft] to be non-inferior in terms anti-PRP antibody response compared to both DTPw-HBV/Hib vaccine and DTPw-HBV/Hib_2.5 _vaccine after a three-dose primary vaccination course. However, the anti-PRP GMC point estimate for the comparator DTPw-HB/Hib_2.5 _vaccine (which, unlike the other 2 vaccines [*Tritanrix*(tm)-HepB/*Hiberix*(tm) and *Zilbrix*-Hib(tm)] used in this study, is not a WHO pre-qualified) vaccine [17,18] was significantly lower than for the full-content DTPw-HB/Hib. The results from this study agree with previous findings from the Philippines and Latina comparing a DTPw-HB/Hib_2.5 _vaccine with the licensed vaccine [6,19] and support the observation that vaccines containing reduced amounts of PRP-tetanus toxoid conjugate generate effective antibody responses and immunological protection [16,7,20-26]. The responses to the other antigens of DTPw-HBV/Hib_2.5 _[Kft] were also found to be within similar 95% confidence intervals as the other vaccines, in terms of seroprotection and vaccine response, further substantiating the non-inferiority of the DTPw-HBV/Hib_2.5 _[Kft] vaccine to the two comparator vaccines.

Overall, the safety profile of the new DTPw-HBV/Hib_2.5 _[Kft] vaccine appeared to be similar to that of the licensed comparator vaccines and the incidence of symptoms reported was within the range of incidences reported in the literature after DTPw-based combination vaccines [5].

This study was designed to assess primary vaccination at 6, 10 and 14 weeks. In clinical practice an additional booster dose of the vaccine is recommended at 12-18 months. Although a booster was not included in this study, the vaccine was immunogenic (seroprotection above accepted minimum cut-off values) following the primary vaccination course. The non-inferiority analysis was not performed on the PRP cut-off ≥1.0 μg/mL, as the study was not powered for this comparison, although this concentration is regarded as an indicator of long-term protection. Nevertheless, the CIs for the percentage of subjects seroprotected overlapped (at least 94.1% in all groups) indicating that all three vaccines conferred long-term protection. In addition, The Hib immune response to the DTPw-HBV/Hib_2.5 _[Kft] was similar to that observed with another, WHO prequalified, DTPw-HBV-Hib vaccine administered in a 2-3-4 month schedule [27].

Reduction of the antigen content of the Hib component of DTPw-HBV/Hib and the different formulation of the DTPw-HBV/Hib_2.5 _[Kft] DTPw antigens did not have any significant effects on the safety, reactogenicity and immunogenicity parameters tested of the combination vaccine when compared to the licensed comparator vaccines. However, further studies need to be carried out to assess any interference on other vaccines, which may be co-administered at the EPI time points, such as rotavirus vaccine or conjugated pneumococcus vaccine.

## Conclusions

The reduced PRP, new formulation, DTPw-HBV/Hib_2.5 _[Kft] vaccine was immunogenic and well tolerated when administered according to the EPI schedule to Indian infants. It was also shown to be non-inferior to DTPw-HBV/Hib and DTPw-HBV/Hib_2.5 _in terms of seroprotection rates to diphtheria, tetanus and HepB antigens and of vaccine response to the pertussis component.

## Abbreviations

ATP: (according to protocol); BPT: (*Bordetella Pertussis*); CI: (confidence interval); DTPw: (diphtheria-tetanus-pertussis); EPI: (Expanded Programme for Immunization); GMC: (geometric mean concentration); GSK: (GlaxoSmithKline); HBsAg: (hepatitis B surface antigen); HBV: (hepatitis B vaccine); Hep B: (hepatitis B); Hib: (Haemophilus influenza type b); IAP: (Indian Academy of Pediatrics); IU: (international unit); rHBsAg: (recombinant HBsAg); PRP: (polyribosyl-ribitol-phosphate); Pw: (whole cell pertussis); SAE: (serious adverse events); WHO: (World Health Organization)

## Competing interests

GlaxoSmithKline Biologicals was the funding source and was involved in all stages of the study conduct and analysis. GSK Biologicals also funded all costs associated with the development and the publishing of the present manuscript.

Sanjoy Datta, Jeanne-Marie Jacquet and Alix Collard are all employed by GlaxoSmithKline.

## Authors' contributions

The corresponding author had full access to the data and was responsible for submission of the publication.

All authors participated in the design or implementation, analysis and interpretation of the study, the writing of the report and the decision to submit the manuscript for publication.

## Pre-publication history

The pre-publication history for this paper can be accessed here:

http://www.biomedcentral.com/1471-2334/10/298/prepub

## References

[B1] Decker MD Principles of pediatric combination vaccines and practical issues related to clinical practice Pediatr Infect Dis J 2001 20 11 Suppl S10 18 11704718 10.1097/00006454-200111001-00002

[B2] Kalies H Grote V Verstraeten T Hessel L Schmitt HJ von Kries R The use of combination vaccines has improved timeliness of vaccination in children Pediatr Infect Dis J 2006 25 507 12 10.1097/01.inf.0000222413.47344.23 16732148

[B3] World Health Organization and UNICEF Global immunization coverage 2009 http://www.who.int/immunization/newsroom/GID_english.pdf Last accessed April 1st 2009

[B4] Chunsuttiwat S Biggs BA Maynard JE Thammapormpilas P O-Prasertsawat M Comparative evaluation of a combined DTP-HB vaccine in the EPI in Chiangrai Province, Thailand Vaccine 2002 21 188 93 10.1016/S0264-410X(02)00461-9 12450693

[B5] Arístegui J Usonis V Coovadia H Riedemann S Win KM Gatchalian S Bock HL Facilitating the WHO expanded program of immunization: the clinical profile of a combined diphtheria, tetanus, pertussis, hepatitis B and Haemophilus influenzae type b vaccine Int J Infect Dis 2003 7 143 51 10.1016/S1201-9712(03)90011-7 12839717

[B6] Gatchalian S Reyes M Bernal N Lefevre I David MP Han HH Bock HL Wolter J Schuerman L A new DTPw-HBV/Hib vaccine is immunogenic and safe when administered according to the EPI (Expanded Programme for Immunization) schedule and following hepatitis B vaccination at birth Human Vaccines 2005 1 198 203 17012860 10.4161/hv.1.5.2163

[B7] Lagos R Valenzuela MT Levine OS Losonsky GA Erazo A Wasserman SS Levine MM Economisation of vaccination against Haemophilus influenzae type b: a randomised trial of immunogenicity of fractional-dose and two-dose regimens Lancet 1998 351 9114 1472 6 10.1016/S0140-6736(97)07456-4 9605803

[B8] ICMR Indian Council of Medical Research guidelines governing clinical trials 2006 http://icmr.nic.in/ethical_guidelines.pdf

[B9] World Health Organization Immunization Policy. Global Programme for Vaccines and Immunization, Expanded Programme on Immunization 1996 WHO/EPI/GEN/95.03REV.1

[B10] World Health Organization Introduction of hepatitis B vaccine into childhood immunization services 2001 http://www.who.int/immunization_delivery/publications/HepB-MG_English.pdf Last accessed 1st April 2009

[B11] World Health Organization WHO position paper on *Haemophilus influenzae *type b conjugate vaccines Wkly Epidemiol Rec 2006 81 47 445 52 17124755

[B12] Marshall GS Happe LE Lunacsek OE Szymanski MD Woods CR Zahn M Russell A Use of combination vaccines is associated with improved coverage rates Pediatr Infect Dis J 2007 26 6 496 500 10.1097/INF.0b013e31805d7f17 17529866

[B13] Prymula R Plisek S Clinical experience with DTPw-HBV and DTPw-HBV/Hib combination vaccines Exp Opin Biol Ther 2008 8 4 503 13 10.1517/14712598.8.4.503 18352853

[B14] Bavdekar SB Maiya PP Subba Rao SD Datta SK Bock HL Immunogenicity and safety of combined diphtheria-tetanus-whole cell pertussis-hepatitis B/Haemophilus influenzae type b vaccine in Indian infants previously primed at birth with hepatitis B vaccination Indian Pediatrics 2007 44 7 505 10 17684303

[B15] Bravo L Carlos J Gatchalian S Borja-Tabora C Bibera G Willems P Safary A Bock HL The new DTPw-HBV-Hib combination vaccine can be used at the WHO schedule with a monovalent dose of hepatitis B vaccine at birth Southeast Asian J Trop Med Public Health 1998 29 772 8 10772563

[B16] Hla KH Thein SA Aye A Han HH Bock HL David MP Schuerman L Reactogenicity and immunogenicity profiles of a novel pentavalent diphtheria-tetanus-whole cell pertussis-hepatitis B and *Haemophilus influenzae *type b vaccine: a randomized dose-ranging trial of the Hib tetanus-conjugate content Pediatr Infect Dis J 2006 25 706 12 10.1097/01.inf.0000223488.80814.df 16874170

[B17] World Health Organisation Immunization Standards. Diphtheria-Tetanus-Pertussis (whole cell)-Hepatitis B-Haemophilus influenzae type b (1 dose vial) http://www.who.int/immunization_standards/vaccine_quality/56_diphteta/en/index.html Last accessed 12^th ^July 2010.

[B18] World Health Organisation Immunization Standards. Diphtheria-Tetanus-Pertussis (whole cell)-Hepatitis B-Haemophilus influenzae type b (1 dose vial) http://www.who.int/immunization_standards/vaccine_quality/59_diphteta/en/index.html Last accessed 12^th ^July 2010.

[B19] Espinoza F Tregnaghi M Gentile A Abarca K Casellas J Collard A Lefevre I Jacquet J-M Primary and booster vaccination in Latin American children with a DTPw-HBV/Hib combination: a randomized controlled trial BMC Infect Dis 2010 in press 20950456 10.1186/1471-2334-10-297 PMC2967556

[B20] Fernandez J Balter S Feris J Gomez E Garib Z Castellanos PL Sánchez J Romero-Steiner S Levine OS Randomized trial of the immunogenicity of fractional dose regimens of PRP-T Haemophilus influenzae type b conjugate vaccine Am J Trop Med Hyg 2000 62 485 90 11220764 10.4269/ajtmh.2000.62.485

[B21] Romero-Steiner S Fernandez J Biltoft C Wohl ME Sanchez J Feris J Balter S Levine OS Carlone GM Functional antibody activity elicited by fractional doses of *Haemophilus influenzae *type b conjugate vaccine (polyribosylribitol phosphate-tetanus toxoid conjugate) Clin Diagn Lab Immunol 2001 8 1115 9 11687449 10.1128/CDLI.8.6.1115-1119.2001 PMC96235

[B22] Campbell JD Lagos R Levine MM Losonosky GA Standard and alternative regimens of *Haemophilus influenzae *type b conjugate vaccine (polyribosylribitol phosphate-tetanus toxoid conjugate vaccine) elicit comparable antibody avidities in infants Pediatr Infect Dis J 2002 21 822 6 10.1097/00006454-200209000-00007 12352802

[B23] Huebner RE Nicol M Mothupi R Kayhty H Mbelle N Khomo E Klugman KP Dose response of CRM197 and tetanus toxoid-conjugated *Haemophilus influenzae *type b vaccines Vaccine 2004 23 802 6 10.1016/j.vaccine.2004.06.052 15542205

[B24] Nicol M Huebner R Mothupi R Käyhty H Mbelle N Khomo E Haemophilus influenzae type b conjugate vaccine diluted tenfold in diphtheria-tetanus-whole cell pertussis vaccine: a randomized trial Paediatr Infect Dis J 2002 21 138 41 10.1097/00006454-200202000-00010 11840081

[B25] Tregnaghi M Lopez P Rocha C Rivera L David MP Ruttimann R Schuerman L A new DTPw-HB/Hib combination vaccine for primary and booster vaccination of infants in Latin America Rev Panam Salud Publica 2006 19 179 88 10.1590/S1020-49892006000300006 16640847

[B26] Gatchalian S Reyes M Bermal N Chandrasekaran V Han HH Bock HL Lefevre I A new DTPw-HBV/Hib vaccine: immune memory after primary vaccination and booster dosing in the second year of life Human Vaccines 2008 4 1 60 6 18376148 10.4161/hv.4.1.5069

[B27] Kanra G Kara A Demiralp O Contorni M Hilbert AK Spyr C Viviani S Safety and immunogenicity of a new fully liquid DTPw-HepB-Hib combination vaccine in infants Hum Vacc 2006 2 4 155 60 10.4161/hv.2.4.2942 17012890

